# The Impact of Kidney Stones on Congestive Heart Failure Risk

**DOI:** 10.7759/cureus.62188

**Published:** 2024-06-11

**Authors:** Marc Ganz, Daniel Miller, Jude Appiah, David Sezanayev, Emily Kohanbash, David Khanimov, Andrew Winer

**Affiliations:** 1 Department of Urology, State University of New York Downstate Health Sciences University, Brooklyn, USA; 2 Department of Internal Medicine, Icahn School of Medicine at Mount Sinai, Queens Hospital Center, New York, USA; 3 Department of Neurology, New York Institute of Technology (NYIT) College of Osteopathic Medicine, Old Westbury, USA; 4 Department of Internal Medicine, State University of New York Downstate Health Sciences University, Brooklyn, USA; 5 Department of Emergency Medicine, Xavier University School of Medicine Aruba, Oranjestad, ABW

**Keywords:** epidemiology, nhanes, cardiovascular risk, kidney stones, congestive heart failure

## Abstract

Introduction

Heart failure (HF) represents a substantial global health concern, evidenced by its high prevalence, significant mortality rates, and considerable economic impact worldwide. Within this broader context, congestive heart failure (CHF) emerges as a critical subset, affecting millions and leading to high rates of morbidity and mortality. Recent explorations have started to uncover a potential link between kidney stones and broader systemic health problems, including coronary artery disease. This association suggests that kidney stones might also indicate an increased risk for cardiovascular diseases such as CHF. However, the exploration into the direct relationship between kidney stones and CHF is still in its nascent stages, creating a significant gap in understanding the full cardiovascular implications of kidney stone disease.

Methods

Utilizing data from the National Health and Nutrition Examination Survey (NHANES) for the period of March 2017 to March 2020, we conducted a logistic regression analysis to assess the relationship between kidney stones and CHF. This analysis adjusted for key variables such as age, gender, race, and educational attainment, aiming to isolate the impact of kidney stones on CHF risk among 8,521 participants.

Results

Our findings revealed a higher incidence of CHF among individuals with a history of kidney stones (7%) compared to those without (3%). Logistic regression analysis further highlighted kidney stones as an independent risk factor for CHF, with an odds ratio (OR) of 1.857, significant at p < 0.01. These results underline the importance of considering kidney stones in the broader context of cardiovascular health risks, particularly CHF, as their presence significantly elevates the risk compared to the general population without kidney stones. Additional demographic analyses indicated significant influences of age, gender, race, and educational level on the risk of CHF, emphasizing the complex interplay between these factors and heart health.

Conclusion

The study confirms the association between a history of kidney stones and an increased risk of CHF, suggesting the need for heightened cardiovascular monitoring for patients with such a history. It also brings to light the significant role demographic factors play in CHF risk, advocating for targeted interventions to mitigate these disparities. Our research supports a broader view of patient care that includes consideration of urological conditions as potential risk factors for heart failure. Further exploration into the mechanisms linking kidney stones and cardiovascular health is recommended to inform more effective prevention and treatment strategies.

## Introduction

Heart failure (HF) imposes a significant global burden, characterized by a prevalence rate of 1%-3% in the adult population worldwide, an incidence rate of 1-20 cases per 1,000 individuals, and a five-year mortality rate of 50%-75% [1]. The estimated economic cost associated with heart failure stands at $30.7 billion [2]. The prevalence and incidence rates, combined with the significant mortality rate linked to heart failure (HF), indicate that congestive heart failure (CHF), as a specific category of HF, probably carries a comparable burden. In fact, CHF is recognized globally as a prevalent condition with high rates of morbidity and mortality, affecting an estimated 26 million people worldwide [3].

Recent literature has increasingly identified kidney stones as not merely an isolated urological condition but as a marker potentially indicative of broader systemic health issues. Conditions such as hypertension, metabolic syndrome, diabetes, gout, and chronic kidney disease have all been associated with a history of kidney stones [4], suggesting a complex interplay between kidney stone formation and systemic health dysfunctions. This expanding body of research also explores the relationships between kidney stone formers and an accumulation of risk factors for coronary artery disease (CHD), indicating a potential vascular component to the kidney stone disease process [5-7].

The direct relationship between kidney stones and congestive heart failure (CHF) remains markedly less developed, and the evidence directly exploring this potential relationship remains sparse, creating a significant gap in understanding the full cardiovascular implications of kidney stone disease. While several studies have identified kidney stones as a potential risk factor for CHD, suggesting a broader vascular pathology, others have shown no significant correlation, highlighting the need for more rigorous and detailed research [8-10]. This complexity underscores the importance of a deeper investigation into how kidney stones could contribute to cardiovascular risk, particularly CHF, and points toward an intricate interplay that warrants further exploration to fully understand the cardiovascular sequelae of kidney stone disease.

## Materials and methods

Data collection

Our study analyzed data provided by the National Health and Nutrition Examination Survey (NHANES), a project administered by the CDC. The dataset we analyzed covers the period from March 2017 to March 2020. These cycles were chosen because they contain the most recent and relevant data regarding the medical conditions under investigation. The dataset features detailed responses from participants, focusing specifically on two sections within the NHANES dataset: "medical conditions" and "kidney conditions-urology." The former includes valuable information on congestive heart failure, while the latter offers insights into the prevalence and details of kidney stones. These sections represent a structured breakdown within NHANES, allowing for targeted analysis of these health issues. The study's protocol received approval from the Review Board of the Physician's Journal of Medicine, based in Queens, New York, USA, under the approval number F2024051.

Variables

The inclusion criteria focused on participants' medical histories regarding these conditions. The primary independent variable was the history of kidney stones, determined by a "yes" response to the survey question, "Have you ever had kidney stone disease?" The dependent variable was a history of CHF, established through an affirmative response to the question, "Has a doctor or other health professional ever told you that you had congestive heart failure?"

Participants who answered "no" to either question were excluded from the study. To account for influential variables, adjustments were made for factors such as gender, age, race, educational attainment, and marital status. These factors were selected based on prior research indicating their potential impact on both kidney stone prevalence and cardiovascular health outcomes. This careful selection aims to mitigate confounding and ensure that the observed associations are as robust and meaningful as possible. This detailed logistic regression analysis aimed to precisely evaluate the relationship between kidney stones and the likelihood of having a history of congestive heart failure.

Statistical analysis

R was used to analyze data from the NHANES database. Descriptive statistics were computed to depict the demographic and health characteristics of the cohort, such as average gender, age, racial composition, levels of education, and marital status, all categorized by kidney stone history. The investigation progressed to fitting a logistic regression model to examine the relationships between CHF status and a variety of predictors. This step aimed to shed light on the connection between having a history of kidney stones and the likelihood of experiencing congestive heart failure, alongside other demographic and health factors. The analysis yielded outcomes like odds ratios, confidence intervals, and p-values, which were evaluated for significance. We set the significance level at p < 0.05 for all analyses. This comprehensive statistical evaluation was carried out using R version 4.2.0 (Released in 2023 by the R Foundation for Statistical Computing, based in Vienna, Austria).

## Results

In our study of 8,521 participants, 812 reported a history of kidney stones, and 7,709 did not. The median age of participants with kidney stones was 55.87, compared to 50.47 for those without. The incidence of CHF was 58 (7%) among those with a history of kidney stones, higher than the 264 (3%) observed in participants without kidney stones.

Demographic analyses revealed significant variations. The prevalence of CHF among different races showed that 386 (48%) participants with kidney stones were White, compared to 2,540 (33%) in the group without a history of kidney stones; 133 (16%) were Black, compared to 2,155 (28%) without a history of kidney stones; and 83 (10%) were Mexican American, slightly less than the 898 (12%) in the group without a history of kidney stones. Education levels varied, with 300 (37%) kidney stone patients having some college or an associate degree, compared to 2,468 (32%) in the group without a history of kidney stones. Concerning marital status, 490 (60%) of those with a history of kidney stones were married, versus 4,418 (57%) of those without a history of kidney stones (Table 1).

**Table 1:** Demographic and Health Characteristics of Participants by Kidney Stone History CHF: congestive heart failure.

Variable	History of Kidney Stones N (%)	No History of Kidney Stones N (%)
Total	812	7,709
Age (median)	55.87	50.47
CHF	58 (7%)	264 (3%)
Race	-	-
Mexican American	83 (10%)	898 (12%)
Other Hispanic	99 (12%)	781 (10%)
White	386 (48%)	2,540 (33%)
Black	133 (16%)	2,155 (28%)
Multi-racial	111 (14%)	1,335 (17%)
Education level	-	-
Less than 9th grade	60 (7%)	599 (8%)
9th-11th grade	101 (12%)	844 (11%)
High school	174 (21%)	1,876 (24%)
Some college	300 (37%)	2,468 (32%)
College graduate or above	176 (22%)	1,911 (25%)
Marital status	-	-
Married	490 (60%)	4,418 (57%)
Widowed/divorced/separated	219 (27%)	1,727 (22%)
Never married	103 (13%)	1,556 (20%)

Logistic regression analysis revealed that a history of kidney stones significantly increased CHF risk (OR = 1.857). Age was another crucial factor (OR = 1.061), suggesting a gradual rise in CHF risk with age. Gender analysis showed that females had a lower OR of 0.687 for CHF, indicating a protective effect against the development of CHF compared to males. Educational achievement was inversely related to CHF risk, with some college education (OR = 0.784) and a college degree or higher (OR = 0.292) showing an even more substantial protective effect. Racial analysis indicated higher CHF risks for Non-Hispanic Whites (OR = 2.234) and Non-Hispanic Blacks (OR = 2.801), underscoring the need for targeted interventions in these populations. Marital status also influenced CHF risk, with those who were once married (widowed, divorced, or separated) having an OR of 1.326, indicating a higher risk compared to currently married individuals. Interestingly, those who were never married had an OR of 1.256, but this was not statistically significant (Table 2).

**Table 2:** Logistic Regression Analysis of Congestive Heart Failure Risk Factors CI = confidence interval. ***p < 0.001 (highly significant).

Variable	Odds Ratio	Lower CI	Upper CI	P value
Stones	1.857	1.409	2.645	<0.0001***
Age	1.061	1.05	1.073	<0.0001***
Female	0.687	0.505	0.872	0.002013
9th-11th grade	0.948	0.618	1.724	0.828738
High school graduate	0.916	0.592	1.431	0.70032
Some college	0.784	0.504	1.224	0.283808
College graduate or above	0.292	0.169	0.499	<0.0001***
Other Hispanic	1.652	0.773	2.24	0.134657
Non-Hispanic White	2.234	1.256	3.973	0.006294
Non-Hispanic Black	2.801	1.571	4.608	<0.0001***
Other Race, including multi-racial	1.784	0.615	3.394	0.077772
Once married	1.326	1.146	1.714	0.031485
Never married	1.256	0.686	1.89	0.276418

## Discussion

Our study has identified several key demographics, lifestyle, and socioeconomic factors that influence the risk of CHF, including age, gender, history of kidney stones, educational attainment, and racial background. Our analysis confirms the well-established correlation between age and CHF risk, with our results showing a gradual increase in CHF risk with advancing age (OR = 1.061). This is consistent with previous studies that have highlighted age as a significant predictor of CHF [11], likely due to the accumulation of cardiovascular risk factors over time and age-related decline in cardiac function. Additionally, we found an association between kidney stones and an increased risk of CHF (OR = 1.857) in our cohort. This finding adds to a growing body of evidence suggesting a link between urological conditions and cardiovascular health. The link between kidney stones and CHF may be partly explained by underlying metabolic abnormalities, such as hypertension and chronic kidney disease, which are common in individuals with kidney stones and are known risk factors for CHF.

Our findings related to gender differences, with females having a lower odds of CHF (OR = 0.687), align with previous reports that indicate men may have a higher risk of CHF at earlier stages of life, although the risk tends to equalize with advancing age. This gender disparity has been attributed to hormonal differences in between genders [12].

Educational attainment emerged as a protective factor against CHF, particularly for those with college education or higher (OR = 0.292). This supports the hypothesis that higher socioeconomic status, often associated with better education, affords individuals access to healthier lifestyles, better healthcare, and reduced exposure to stressors known to contribute to cardiovascular risk [13,14].

The racial disparities observed, with Non-Hispanic Whites (OR = 2.234) and Non-Hispanic Blacks (OR = 2.801) having higher odds of CHF, mirror longstanding discussions in the literature about the impact of race and ethnicity on health outcomes [15-17]. These disparities can be attributed to a complex interplay of genetic, environmental, socioeconomic, and healthcare-related factors. Notably, our findings emphasize the heightened risk among Non-Hispanic Blacks, underscoring the urgent need for targeted interventions to address the social determinants of health that disproportionately affect this population.

Interestingly, marital status was also associated with CHF risk, where those who were once married had slightly higher odds of CHF (OR = 1.326). While less commonly studied, marital status may reflect underlying social support systems, stress levels, and health behaviors, which can contribute to cardiovascular health outcomes.

Limitations

This study's limitations include its reliance on cross-sectional data from NHANES, which restricts our ability to establish causality between kidney stones and CHF. Additionally, the use of self-reported data may introduce recall bias and misclassification of conditions, potentially affecting the accuracy of the findings. Although adjustments were made for various demographic and socioeconomic factors, residual confounding by unmeasured variables such as lifestyle behaviors and genetic factors could influence the observed associations. Furthermore, the specific population sampled in NHANES may limit the generalizability of these findings to other groups.

The cross-sectional design prevents us from confirming the temporal sequence between kidney stones and CHF, which could influence interpretations of causality. Moreover, the generalizability of our results is limited primarily to the U.S. population and may not fully apply to international contexts with different health profiles and healthcare systems. Finally, this study does not explore the underlying biological mechanisms that could explain the relationship between kidney stones and CHF, highlighting the need for further clinical and experimental research to clarify these potential causal pathways.

## Conclusions

In conclusion, our study has highlighted significant associations between various factors and the risk of congestive heart failure (CHF), revealing the complex interplay of genetic, environmental, and socioeconomic influences on cardiovascular health. Notably, age, gender, history of kidney stones, educational attainment, and racial background were all found to impact CHF risk, providing valuable insights for future research and public health strategies.

Our findings emphasize the need for closer cardiovascular monitoring in patients with a history of kidney stones due to their increased CHF risk and highlight gender-specific health strategies to address differing CHF risks. The protective effect of higher education levels on CHF risk underscores the importance of socioeconomic factors in health outcomes, while the observed racial disparities call for targeted public health interventions. By reinforcing known associations and uncovering new links, our study advocates for a comprehensive approach to CHF prevention that addresses individual, societal, and environmental risk factors. Future research should focus on the underlying mechanisms of these associations, particularly the connection between kidney stones and cardiovascular health, to develop targeted prevention and treatment strategies and mitigate the impact of socioeconomic and racial disparities on CHF risk. To elucidate the mechanisms behind the association between kidney stones and CHF, future studies should focus on longitudinal and mechanistic research to clarify causal relationships and underlying biological pathways.
